# A method to implement continuous characters in digital identification keys that estimates the probability of an annotation

**DOI:** 10.1002/aps3.1247

**Published:** 2019-05-08

**Authors:** Christopher D. Tyrrell

**Affiliations:** ^1^ Botany Department Milwaukee Public Museum 800 W. Wells Street Milwaukee Wisconsin 53233 USA

**Keywords:** continuous, identification, key, morphology, naive Bayesian classifier

## Abstract

**Premise:**

Species identification is vital to many disciplines. Digital technology has improved identification tools, but the direct use of characters with continuous states has yet to be fully realized. To achieve full use of continuous characters for identification, I propose a classifier that calculates a posterior probability (degree of belief) in possible name assignments and an estimate of the relative evidence for the candidate annotations.

**Methods:**

A model for a species is defined using continuous morphological characters, and an algorithm for identification with a naive Bayesian classifier, using the model, is presented. A method of estimating the strength of evidence for candidate species is also described.

**Results:**

The proposed method is applied in two example identifications: native vs. invasive *Myriophyllum* in North America and vegetative *Rhipidocladum* bamboos in Mexico. In each instance, the new method provides a probability and estimate of the strength of the probability to enhance the name assignment in situations where taxa are difficult to differentiate using discrete character states.

**Discussion:**

Naive Bayesian classifiers take advantage of the predictive information inherent in continuous morphological characters. Application of this methodology to plant taxonomy advances our ability to leverage digital technology for improved interactive taxonomic identifications.

Assigning a species identity to an organism is a fundamental and essential activity in a wide array of disciplines including ecology, conservation, forestry/agriculture, fisheries, and land use planning and assessment (Lyal et al., 2011; Barrows et al., 2016). The idea of what a species is, conceptually and operationally, is a topic with a rich philosophical debate and history (see Mayden 1997 and Wilkins 2009 and references therein). In practice, most identifications of plant species are made based on morphology using phenotypic characters. The methods used to assign identifications to an organism vary from matching of overall morphology (gestalt/rote or general impressions of size and shape) to a systematic, hierarchical descent through technical single‐access keys (Voss, 1952; Radford et al., 1976). When used by a non‐expert, these two methods can form a continuum between speed (gestalt) and accuracy (keys).

Species concepts and morphological identifications, however, are not always straightforward and, when constrained to analog approaches like single‐access dichotomous keys, can lead to complex and convoluted identification manuals or, in rare cases, keys that are truncated to a cluster of similar species (e.g., McClure, 1973: 104). Advances in digital technology offer new platforms to develop multi‐access identification tools and techniques, and these are helping to overcome some of the challenges. Dozens of software tools have been developed over the past 25 years: Intkey (http://www.delta-intkey.com/; Dallwitz, 1980), LucID (http://www.lucidcentral.com), MEKA (http://ucjeps.berkeley.edu/meacham/meka/), NaviKey (http://www.navikey.net/), WEBiKEY (https://github.com/WEBiKEY/InteractiveKey), and many more (see Leggett and Kirchoff [Link] for additional examples). Many of these tools are web‐based, but some have matured into sophisticated stand‐alone applications. Digital platforms can even negate the gestalt–technical key trade‐off by combining the approaches (Jones, 2013). Machine learning technologies such as image recognition (for instance iNaturalist [https://www.inaturalist.org/]) offer an alternative approach to identifications. The author and user of these keys have a more passive role in the process. The author feeds a large number of vetted training images to a sophisticated statistical algorithm to create an identification model. The model then delivers putative identifications to the user based on an input image. With this methodology, it is difficult to determine or reconstruct the actual features or characteristics the algorithm uses to differentiate species.

A limitation of nearly all character‐based digital identification tools is their reliance on discrete states (binary or multistate) to encode continuous characters. Traditional single‐access dichotomous keys put forward a series of static decision‐points. At each point, descendant couplets or taxa are encapsulated into one of two discrete character states. Multi‐access keys improve upon this by using a taxon‐by‐character matrix where each element can contain one or more discrete states. The matrix approach increases the flexibility of multi‐access over single‐access keys because a taxon can have multiple states for a character and the decision‐points of the key can be dynamically generated or modified. Character states in the multi‐access matrix are still formed into discrete units, even for characters with continuous states. This dependence on discrete states is unfortunate because many salient features for identification of a macroscopic plant or animal specimen are composed of characteristics with a continuous nature, and continuous data can offer more refined information than can be achieved using discrete states.

For example, if a couplet in a dichotomous key posits that a plant has a calyx less than 1 cm in length vs. calyx greater than or equal to 1 cm, the two discrete states would be, state 1: calyces are <1 cm, and state 2: calyces are ≥1 cm. Descendant taxa must be categorized into one of these two binary states. Yet, because calyx length is a continuous characteristic, a taxon may have a few individuals that have lengths outside the binary state for which its taxon is encoded. For example, a taxon categorized as state 2 (≥1 cm) might have a few individuals that have 0.9‐cm‐long calyces.

The subsequent pathways from a couplet in a dichotomous key often encapsulate more than one taxon, and additional characters are usually used to discriminate among those taxa. However, when more than one taxon is grouped together, the actual frequency of states for a continuous character like calyx length may follow a complex multimodal form (the actual distribution of calyces in state 2, for instance, might have three modes around 1.2, 2.7, and 5.1 cm). The descendant taxa of state 2 might all or mostly be separable based on subsequent splits of calyx length, but this repeated splitting is inefficient and can be frustrating for a key user.

A multi‐access version of this hypothetical example could achieve more efficient discrimination by categorizing calyx length into four states: state 1, length ≤0.9 cm; state 2, length = 1.0–1.5 cm; state 3, length = 1.6–3.0 cm; and state 4, length >3.0 cm (Table 1). Conflict can still arise, however, if taxa have overlapping ranges (e.g., species B, C, and E in Table 1), thus requiring additional characters to distinguish among them.

**Table 1:** Hypothetical encoding scheme for calyx length in a single‐access (binary) vs. multi‐access (multistate) key.^a^

Taxon	Calyx length range (cm)	Calyx length (binary)	Calyx length (multistate)
Species A	0.7–0.9	1	1
Species B	0.7–1.0	1	1, 2
Species C	0.9–1.5	2	1, 2
Species D	1.7–2.7	2	3
Species E	1.2–4.4	2	2, 3, 4
Species F	3.3–5.1	2	4

a Binary states, 1: <1 cm, 2: ≥1 cm; multistate, 1: ≤0.9, 2: 1.0–1.5, 3: 1.6–3.0, 4: >3.0.

A more robust treatment for overlapping character ranges can be achieved using a probabilistic framework. Keys for microbiological cultures were early adopters of probabilistic identifications (Beers and Lockhart, 1962; Willcox et al., 1980), but these microbial keys did not seem to use, or had limited application of, continuous characters. In fact, the direct use of continuous characters without discretizing the character states has not been widely implemented in any microbiological or macrobiological identification key, despite this being put forward as a desirable feature for digital, interactive keys (Dallwitz, 1994). In this paper, I propose applying a naive Bayesian (NB) classifier to calculate the posterior probability of the name assignment for each candidate taxon in a key. The taxa in the classifier will be modeled using continuous‐normal morphological characters, but the method can be extended to categorical or mixed models. I also adapt a method to estimate the evidence or strength of the probability for each possible name assignment relative to the alternative taxa in the key. I demonstrate the application of this method in two simple examples. My objective is to describe the method and provide developers of web and stand‐alone applications and/or biodiversity scientists who have programming skills a way to integrate this technique into existing digital identification tools or inspire them to develop new software that incorporates the method. To this end, I describe a general algorithm for the method's implementation and provide a simple example script. Secondarily, I hope to raise awareness among revisionary plant taxonomists or floristics researchers of what is possible (so they may seek new features in applications), but also what is required of them to fully leverage the power of continuous morphological characters (i.e., larger sample sizes and reporting summary statistics for characters rather than just character state ranges).

## METHODS

### Morphological model of a species

I assume a species is a hypothetical class to which individuals or specimens can be assigned. Each class consists of morphological characteristics that can be continuous or categorical. The likelihood that an individual of a species takes on a particular state of a character can be described with a probability density or mass function (PDF/PMF). Characters could take on any appropriate PDF or PMF form; a continuous character might have character states that follow a normal PDF, whereas a categorical character might follow a uniform discrete PMF.

As an example, assume some continuous random character is normally distributed for all species. The relative likelihood of an individual in a species class having a measurement (*m*) for a character can be calculated by knowing the mean (μ) and standard deviation (σ) for that character:

(1) $$PD\left(m\;|\;\mu ,\sigma \right)=\frac{1}{\sqrt{2\pi \sigma^{2}}}e^{-\frac{{\left(m-\mu \right)}^{2}}{2\sigma^{2}}}$$

The relative likelihood is used because the absolute likelihood (area under the PDF) is zero for an exact measurement of a continuous random variable. Relative likelihoods can be calculated for all classes in a set of candidate species, and the proportion of any one species class relative to all the others in the candidate set is:

(2) $$p\left(m\;|\;{\text{species}}_{1},\ldots ,\;{\text{species}}_{k}\right)=\frac{PD(m\;|\;{\text{species}}_{i})}{\sum_{j=1}^{k}PD\left(m\;|\;{\text{species}}_{j}\right)}$$

where *k* is the number of candidate species classes. Imagine two potential species classes both with variance = 1, but differing in their means. Assume species A has mean = 4 and species B has mean = 6. If the character measured on a specimen of unknown class is equal to 3, then the relative likelihood that that specimen belongs to species A is 0.242 and to species B is 0.004. The proportion attributable to each species class, then, is species A: 0.242 / (0.242 + 0.004) = 0.98 and species B: 0.004 / (0.242 + 0.004) = 0.02.

Any number of characters can be used in the definition of the species classes. Each of these can be represented geometrically as an axis of a multi‐dimensional morphological character space. Each species class would exist in this “morphospace” as a multidimensional probability cloud. The cloud for a species class would be most dense (and most probable) at the point or region where the states for every character have their maximal or modal probability densities. The shape of the cloud would be determined by the cumulative PDFs or PMFs of the constituent characters.

### Naive Bayes classification

Simple Bayesian networks are used as classifiers for assigning an unknown something to various classes based on information about the unknown and class descriptors. Here, the case is an unknown organism being assigned a taxonomic identification. To simplify a Bayesian network, all class descriptors (characters) can be assumed to be independent of one another or “naïve” (Jensen and Nielsen, 2007). This naive Bayes assumption is unrealistic for all features of biological specimens (characters such as size and mass, for instance, are nearly always correlated), but framing the identification process as an NB classifier allows for incorporation of uncertainty and an estimate of the belief in the classification.

In machine learning, an NB classifier is a supervised learning technique that uses a set of training data to parameterize a model and that model is then applied to real (test) data. Here, I suggest allowing the author of a taxonomic key to develop and parameterize the multiple‐character model for each species using specimen data (or expert opinion). The classifier would then leverage Bayes’ theorem,

(3) $$p\left({\text{species}}_{i}\;|\;m\right)=\frac{p\left(m\;|\;{\text{species}}_{i}\right)\;p\left({\text{species}}_{i}\right)}{p(m)}$$

to calculate a posterior probability of each species given measurements for each character. The term *p*(*m*) is invariant across all species classes and can be ignored (Webb et al., 2005). Thus, the probability of a species class given a measurement is proportionate to the product of the relative likelihood of a measurement, given the species class, and the (prior) probability of the species class. Initially, the prior probability, *p*(species_*i*_), would be 1/*k*, where *k* is the number of competing species in the classifier and *p*(*m* | species_*i*_) would be governed by the PDF or PMF model set by the key author for each character. Multiple characters are incorporated by iteration, recalculating the degree of belief using the posterior probability from the previous iteration as the prior probability in the current iteration. This condenses to:

(4) $$p\left({\text{species}}_{i}\;|\;m_{1},m_{2},\ldots m_{n}\right)\propto p\left({\text{species}}_{i}\right)\prod_{j=1}^{n}p\left(m_{j}\;|\;{\text{species}}_{i}\right)$$

Due to computational limitations of multiplying many small probabilities, summing the log probabilities is recommended for actual implementation,

(5) $$\begin{array}{cc} & \log p\left({\text{species}}_{i}\;|\;m_{1},m_{2},\ldots m_{n}\right)\propto \text{log}\;p\left({\text{species}}_{i}\right)+\\ & \sum_{i=1}^{n}\text{log}\;p\left(m_{j}\;|\;{\text{species}}_{i}\right) \end{array}$$

Using the relative likelihood (Equation (1)) assumes a key user is making perfect measurements or counts or that a specimen is invariant. This assumption can be overcome by attributing some small error to the measurement. There is often an objective estimate for measurement precision. For example, mass measurements are usually performed using an imperfect analytical balance with a manufacturer‐stated precision. A mass measurement of 3.1 mg on a balance with ±0.05 mg precision, gives the interval [3.05, 3.15]. This small error in measurement introduces convenient boundaries for computing a definite integral under a PDF to determine an absolute likelihood. Using the absolute likelihood allows the key user to specify and incorporate the error of or their certainty in their measurement.

Some PDFs and PMFs will result in extreme probabilities of zero. If the joint probabilities are multiplied, an extreme probability of zero voids the other probabilities in the classifier and classification fails. To overcome this limitation, a general additive smoother (Manning et al., 2008: 260) could be used. The NB process described above is outlined in Appendix 1, and an example in R (R Foundation, 2018) is provided in Appendix S1.

### Jaynes evidence

The optimal species class label to be assigned to an unknown specimen is that class with the maximum a posteriori probability (MAP). Using the MAP provides the best name assignment given the model and measurement(s), but it can be difficult to interpret the strength of the classification when two or more classes have posterior probabilities very close to the class with the MAP. To estimate the strength of a determination, I propose using the ratio of the probability that the unknown is a member of a particular class, over the probability that it is not a member of that class. For ease of human interpretability, Jaynes (2003) recommends using 10 times the base‐10 logarithm of this ratio so that the resulting value is expressed in decibels (dB). This “Jaynes evidence” value corresponds to understandable odds where 0 dB of evidence provides 1 : 1 odds, or just as much information for as against the particular identification. Negative evidence points to unfavorable odds in an assigned name (despite it possibly having the MAP), whereas positive evidence values are odds in favor of the name assignment. The greater the dB, the better or worse the odds on a non‐linear scale (Table 2). Jaynes (2003) suggests that 1 dB of evidence is approximately the smallest quantity of plausibility that is perceptible to human intuition.

**Table 2:** Comparison of approximate equivalents between Jaynes evidence (dB), odds ratio, and probability (Jaynes, 2003)

dB	Odds	Probability
30	1000 : 1	0.999
10	10 : 1	0.909
3	2 : 1	0.666
0	1 : 1	0.5

## RESULTS

### Example 1: Invasive vs. native North American *Myriophyllum*

*Myriophyllum spicatum* L. is a well known invasive aquatic plant in North American lakes and waterways. The threat it poses to aquatic ecosystems in the United States and Canada is documented, and enormous sums are spent on prevention, control, and mitigation of its spread (Les and Mehrhoff, 1999; Kelting and Laxson, 2010). *Myriophyllum spicatum* is morphologically very similar to a native North American milfoil species, *M. sibiricum* Kom. This similarity can complicate identification, leading to potential false positive reports of the presence of the invasive *M. spicatum* or accidental culling of the native *M. sibiricum*. Traditionally, these two milfoil taxa have been distinguished based on the number of pinnae or leaf segments present (Fernald, 1919; Coffey and McNabb, 1974). Although this character does provide some discrimination, the number of segments in each species overlaps unless a correction for variation in leaf length is made (Moody and Les, 2007). Overlaid on this is the discovery that the two species hybridize and the hybrids possess intermediate numbers of leaf segments (Moody and Les, 2002, 2007). I use this invasive‐native‐hybrid milfoil system to demonstrate the NB classifier because the example is well studied and includes only three taxa with one character.

Moody and Les (2007) enumerated the leaf segments on approximately 109 specimens of *M. spicatum*,* M. sibiricum*, and *M. spicatum* × *M. sibiricum* and verified their identity genetically. They showed that there was some overlap in leaf segment number between the parent species but considerable overlap of the hybrid with its parents (Table 3). If the hybrid is not included in an identification key that uses this character, the overlap means that determinations made using that key can result in misidentifications. The number of pinnae on a leaf is a discrete value, but the character is in‐aggregate for a species. Thus, I treat this character as continuous, meaning fractional pinnae represent an average of a variable number of segments on a specimen.

**Table 3:** Comparison of the ranges in number of pinnae for *Myriophyllum sibiricum*,* M. spicatum*, and the *M. sibiricum* × *M. spicatum* hybrid from selected recent identification keys. Most keys present the couplet as number of pinnae per side of a leaf; the values presented here for these cases have been doubled for comparison with references that report the total number of pinnae per leaf

Reference	*M. sibiricum*	Hybrid	*M. spicatum*
Wilhelm and Rericha, 2017: 756	≤24 [10–26 in text]	NA	>24 [>26 in text]
Scribailo and Alix, 2014	6–18(–24)	16–28	(20–)24–36(–42)
Voss and Reznicek, 2012: 640	10–22(–26)	NA	(26–)28–34(–40)
Haines, 2011: 620	12–24	NA	24–40
Moody and Les, 2007	9–27	23–39	16–39
Crow and Hellquist, 2000: 194	≤22	NA	≥24

**Figure 1:**
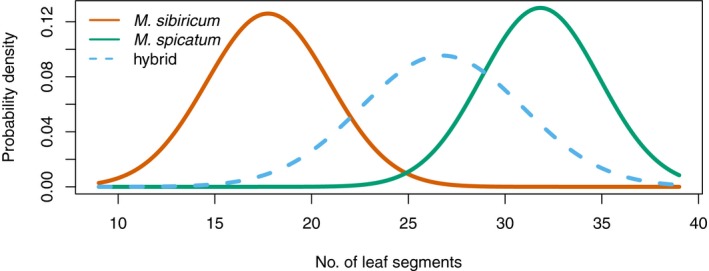
Probability densities for the number of leaf segments in *Myriophyllum spicatum* (solid green), *M. sibiricum* (solid orange), and the *M. sibiricum* × *M. spicatum* hybrid (dashed blue). Mean and variance parameters were estimated from data extracted from Moody and Les (2007).

The frequency curves of the number of leaf segments for each taxon in the Moody and Les (2007) data (Appendix 2) visually resemble normal distributions (Fig. 1). Slight deviations from normality should not serious affect the NB classifier. Characters that have more outliers than are expected of normally distributed data (i.e., their distribution has heavy or inflated tails) could substantially influence posterior probabilities. In these cases, the calculated probability would be too low when it was near the upper or lower end of the character's range and too high when near the mean. Quantile‐quantile plots (Appendix S2) can help visualize trends that might affect the reliability of the PDF chosen for a character.

**Figure 2:**
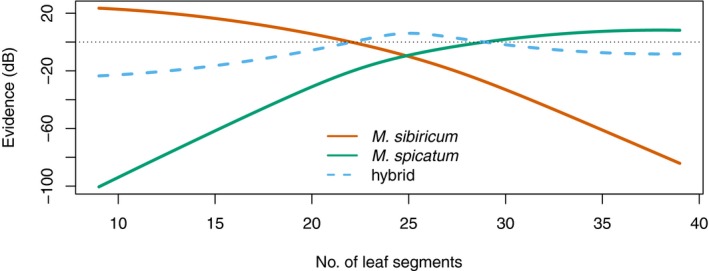
Evidence, 10 times the log ratio of the probability for a species over the probability against a species (Jaynes, 2003), expressed in decibels (dB) along a continuum of leaf segment number for three taxa of watermilfoil: *Myriophyllum spicatum* (solid green), *M. sibiricum* (solid orange), and the *M. sibiricum* × *M. spicatum* hybrid (dashed blue). The dotted black line depicts the point of equal odds; when a curve is above this line, there is positive evidence for species identification. Underlying probabilities were generated from data in Moody and Les (2007).

Because there is only one character in this example, the classifier can only have one iteration, so I proceed directly to a calculation of the Jaynes evidence (Fig. 2). When there are fewer than 22 segments per leaf, there is greater than 1 : 1 odds (positive evidence) that the identity is *M. sibiricum*, and when the number of leaf segments is between 29 and 40 there is greater than 1 : 1 odds that the identity is *M. spicatum*. In the middle (~22–29 segments), the odds favor an identification as the hybrid taxon. At the intersection of the curves near 22 and 29 leaf segments, the evidence is equivocal between the hybrid or one of its parents.

The interpretation of this evidence plot (Fig. 2) is that, in specimens with fewer than 22 segments per leaf, it is more probable than not that an unknown is attributable to *M. sibiricum*. Likewise, when the specimen has more than 29 (but fewer than 40) segments, it is more probable than not that it is attributable to *M. spicatum*. Between 23 and 28 segments per leaf, the evidence for the hybrid exceeds that of either parent species. Specimens with counts near 22 or 29 leaf segments are in a zone of ambiguity where either a parent species or the hybrid may have the MAP, but the evidence for either identification is very weak.

Many dichotomous identification keys to the species of milfoil in North America use the number of leaf segments to distinguish *M. sibiricum* from *M. spicatum* (e.g., Crow and Hellquist, 2000; Haines, 2011; Voss and Reznicek, 2012; Scribailo and Alix, 2014; Wilhelm and Rericha, 2017). All of the keys cited in Table 3 split the two parent species at a value that falls within the region where the hybrid has the best evidence except for Scribailo and Alix (2014). In their key, the dichotomy overlaps the lower ambiguous zone (~22 segments). This imparts a slightly greater chance of falsely identifying *M. sibiricum* or the hybrid as *M. spicatum* when using the Scribailo and Alix key over the others. The posterior probability and Jaynes evidence, in contrast, provide objective, reproducible values to defend a particular taxon name assignment.

### Example 2: Vegetative bamboos in Mexico

In this second example, there are four species of bamboo and two characters. Grasses (Poaceae) are a challenge to accurately identify when in flower, but they are notoriously difficult to identify in a nonflowering condition. The bamboos offer a particularly acute example of this. Of the more than 1300 species of woody bamboo worldwide (Clark et al., 2015), many have long intervals between periods of flowering. The most notable examples are observed in a few species of temperate woody bamboo from Asia where flowering cycles of more than 100 years have been recorded (Janzen, 1976). Many Neotropical species have flowering cycles between 15 and 60 years (Guerreiro, 2014). The net result of this phenology is that most bamboos are encountered or collected in a nonflowering (vegetative) condition when they are hardest to identify to species.

**Figure 3:**
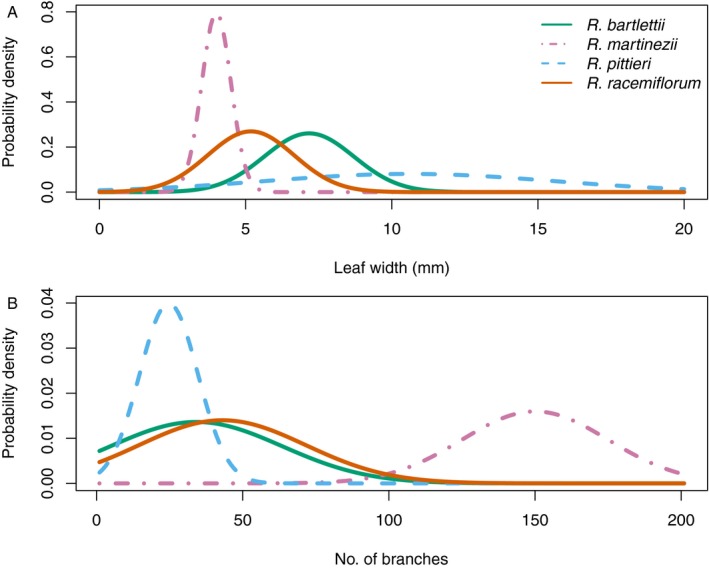
Probability densities for each of four species of Mexican bamboo, *Rhipidocladum bartlettii* (solid green), *R. martinezii* (dash‐dot purple), *R. pittieri* (dashed blue), and *R. racemiflorum* (solid orange), for two characters: (A) foliage leaf width (mm) and (B) number of branches per mid‐culm node.

A recent floristic treatment of the native bamboos of Mexico (Ruiz‐Sanchez et al., 2015) includes keys to the species for several genera. I have selected their key to the species of *Rhipidocladum* McClure for this example because it uses vegetative characters to separate species, I am well acquainted with the genus, and I have quantitative specimen data (Appendix 3) similar to the database of Moody and Les (2007). There are four known species of *Rhipidocladum* in Mexico: *R. bartlettii* (McClure) McClure, *R. martinezii* Davidse & R. W. Pohl, *R. pittieri* (Hack.) McClure, and *R. racemiflorum* (Steud.) McClure. The characters used by Ruiz‐Sanchez et al. are: (1) number of branches at the mid‐culm node, (2) foliage leaf blade width in millimeters, and (3) incidence of fimbriae. I have excluded the fimbriae character from this example. Like leaf segments in the milfoil example, the number of branches is discrete but treated as continuous‐normal here. The number of branches and leaf widths are normally distributed for three of the species (*R. bartlettii*,* R. pittieri*, and *R. racemiflorum*), but *R. martinezii* has too small of a sample size (*n* = 3) to assess its normality (Fig. 3).

**Figure 4:**
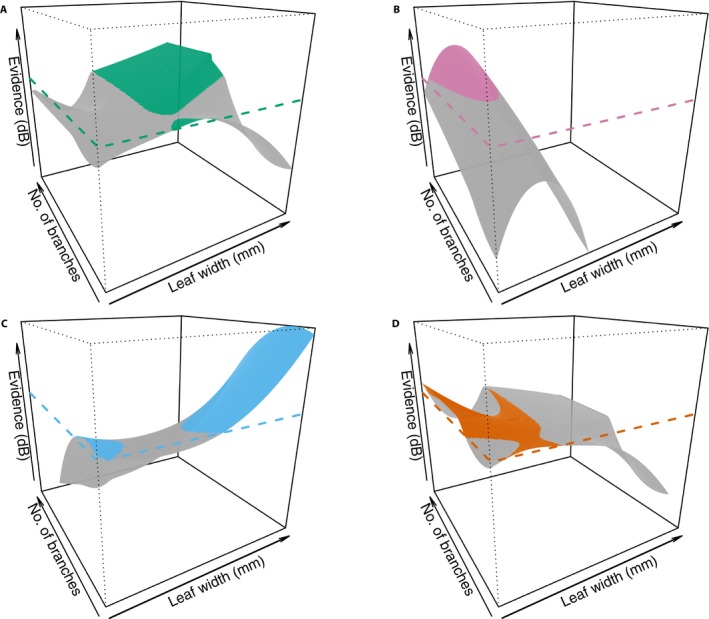
Evidence surfaces, 10 times the log ratio of the probability for a species over the probability against a species (Jaynes, 2003), in decibels (dB) for each of four species of Mexican bamboo across two characters: foliage leaf width (mm) and number of branches per mid‐culm node. (A) *Rhipidocladum bartlettii*, (B) *R. martinezii*, (C) *R. pittieri*, and (D) *R. racemiflorum*. Dashed lines depict the level of equal odds. Areas of the surfaces that lie above the equal odds plane are colored and represent combinations of character states with evidence favoring a taxon's identification.

**Figure 5:**
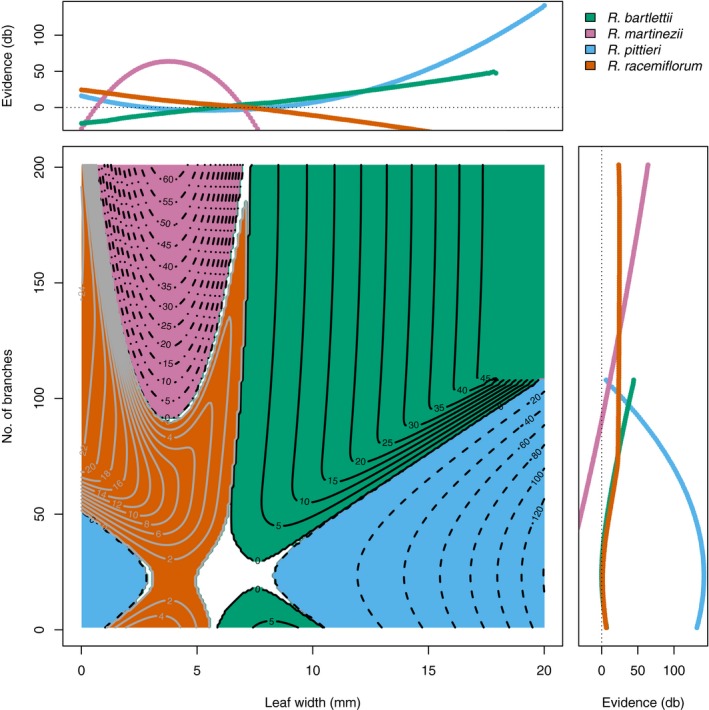
Contours of the odds in favor of a specimen belonging to one of four species of Mexican bamboo (*Rhipidocladum bartlettii* [solid black on green], *R. martinezii* [dash‐dot black on purple], *R. pittieri* [dashed black on blue], and *R. racemiflorum* [solid gray on orange]) plotted across the continuum of states for foliage leaf widths in millimeters (*x*‐axis) and the number of branches per mid‐culm node (*y*‐axis). Marginal plots show the evidence, 10 times the log ratio of the probability for a species over the probability against a species (Jaynes, 2003), in decibels (dB), for each character independently. Line colors in marginal plots conform to their respective species.

Because there are now two characters, the evidence varies over the range of both characters, creating an evidence surface (Fig. 4). The most obvious differences in the evidence among the four species is that *R. bartlettii* and *R. pittieri* (Fig. 4A, C) have relatively flat surfaces, whereas *R. martinezii* (Fig. 4B) and *R. racemiflorum* (Fig. 4D) both have regions of steeply decreasing odds, with the most negative parts of the surfaces following opposing axes (characters). The regions of evidence for each species do not fall into the simple boxes that a species description, which lists only range values, might imply. An examination of the positive evidence only (Fig. 5) shows a complex, mosaic pattern and a region of ambiguity centered around 6‐mm leaf widths and 38 branches per node. Within this zone, none of the four species has good odds of being the best assignment for any specimen.

To examine the models, I used measurements from the type specimens of each of the four species. My measuring error for leaf widths was 0.5 mm and counts I assumed to be ±1.0 branch. For comparison, I calculated the posterior probabilities for each character in isolation and combined (Table 4). Leaf widths alone generated a MAP of the correct species assignment for *R. bartlettii* and *R. martinezii*. Leaf width failed to place the type specimen for *R. pittieri* into the correct species class. In the case of *R. racemiflorum*, leaf widths produced an ambiguous result, returning an equal MAP for either *R. racemiflorum* or *R. bartlettii*. In contrast, number of branches alone recovered the correct species assignment for *R. martinezii* and *R. pittieri*, but not *R. bartlettii*. The *R. racemiflorum* type is fragmentary, and this character could not be counted. When the two characters are both considered, thus iterating the NB classifier, the resulting posterior probabilities for each type specimen are maximal at the correct species for *R. bartlettii* and *R. martinezii*, while *R. racemiflorum* cannot be assessed due to the specimen lacking nodes with branches. *Rhipidocladum pittieri*, however, demonstrates an interesting result. Two species classes had the MAP for the *R. pittieri* type: *R. racemiflorum* (posterior probability = 0.021) and *R. bartlettii* (posterior probability = 0.021), but *R. pittieri* (posterior probability = 0.017) was a relatively close second.

**Table 4:** Posterior probabilities (PP) from the naive Bayesian classifier when considering measurements of leaf width (mm) and number of branches per node from the type specimens of four species of native Mexican bamboo. The three sets of PP represent leaf width alone, number of branches alone, and both characters in combination.^a^

Character and possible species annotation	*Bartlett 12154*	*Martínez S. 19767*	*Tonduz* (*Pittier*) *7193*	*Ghiesbrecht 234*
Leaf width (mm)	7.5	3	6	6.2
*R. bartlettii*	**0.157**	0.006	0.102	**0.111**
*R. martinezii*	<0.001	**0.139**	0.001	<0.001
*R. pittieri*	0.042	0.022	0.028	0.029
*R. racemiflorum*	0.051	0.082	**0.120**	**0.111**
No. of branches	23	135	25	NA
*R. bartlettii*	0.050	0.001	0.050	NA
*R. martinezii*	<0.001	**0.248**	<0.001	NA
*R. pittieri*	**0.156**	<0.001	**0.155**	NA
*R. racemiflorum*	0.043	0.001	0.045	NA
Leaf width combined with no. of branches				
*R. bartlettii*	**0.032**	<0.001	**0.021**	NA
*R. martinezii*	<0.001	**0.138**	<0.001	NA
*R. pittieri*	0.026	<0.001	0.017	NA
*R. racemiflorum*	0.009	<0.001	**0.021**	NA

Note

NA = not applicable.

a Values in bold highlight the most probable species name assignment for each specimen. Specimen measurements are listed on the top row of the set with each character in isolation. Type specimens are identified by their collector, collector number, and herbarium: *Rhipidocladum bartlettii* (McClure) McClure = *Bartlett 12154* (US), *R. martinezii* Davidse & R. W. Pohl = *Martínez S. 19767* (MO), *R. pittieri* (Hack.) McClure = *Tonduz* (*Pittier*) *7193* (US), and *R. racemiflorum* (Steud.) McClure = *Ghiesbrecht 234* (P). Herbarium codes conform to Index Herbariorum (Thiers, 2017).

In the case of *R. pittieri*, the evidence plots can provide some guidance in interpreting the posterior probabilities. At leaf widths = 6 mm (5.5 to 6.5) and 25 (24 to 26) branches per node (the measured values for the *R. pittieri* type specimen), the evidence plot (Fig. 5, contour plot) shows the point (6, 25) to lie within the zone of ambiguity. Any specimens with measurements around these character states will not have good odds of an accurate identification. Looking at the marginal evidence plots of Fig. 5 reveals leaf width (top margin plot) has weak evidence for *R. pittieri* around 6 mm, whereas the number of branches (right margin plot) has strong evidence for *R. pittieri* around 25 branches. A re‐examination of the MAPs for the *R. pittieri* type when each character is considered alone shows the number of branches (where there is strong evidence) correctly assigns the type to *R. pittieri* while leaf width (where there is weak evidence) does not. *Rhipidocladum racemiflorum* provides an example of the method's behavior with missing data. The *R. racemiflorum* type only had leaf widths to use for an identification and the MAP equally assigned the type to both *R. bartlettii* and *R. racemiflorum*. Looking at the evidence plot, the leaf width for the *R. racemiflorum* type specimen (6.2 mm) runs through the zone of ambiguity and follows the line where the *R. bartlettii* and *R. racemiflorum* positive evidence surfaces meet each other. In this case, both assignments remain equally probable.

The type specimen of *R. martinezii* was accurately assigned to the correct species for each character alone and in combination. For *R. bartlettii* and *R. pittieri*, the species class with the MAP probability for the type specimen changed depending on which of the two characters was considered. Combining both characters led to an accurate most probable species class for *R. bartlettii* but not *R. pittieri*. This apparent instability reflects the complexity of continuous morphological characters. The evidence plots reveal the discriminatory power (or lack thereof) for the characters at various points along the continuum of states.

## DISCUSSION

In the two examples presented here, I used a small number of taxa with only one or two characters to make the examples understandable. Having more than two characters is possible and preferable because more characters provide the user with more flexibility and may result in a more stable MAP identification. At four or more characters, each species would have an evidence hypervolume in the appropriate dimensional morphospace. These become impossible to visualize, but they function conceptually similar to the evidence regions in Figures 4 and 5.

In published dichotomous keys, the uncertainty from the author and user of a key enters the identification process in a non‐structured way, so it is not easily quantifiable or replicable. In complex situations, the author of a dichotomous key can attempt to anticipate and minimize user errors by constructing the key with repeated pathways or qualifying information in the couplets to express their uncertainty and mitigate errors in user interpretation. The uncertainty in an identification, however, is latent and arises from a user's choices and comparisons with descriptions. Two users, using the same dichotomous key and set of measurements, may arrive at different results due to their judgments and choices while using the key.

A valuable benefit of digital multi‐access keys is that they provide users with freedom in the choice and order of characters they can use for identification, and better accommodate user uncertainty by allowing a taxon to have multiple character states. If encoded wisely, multi‐access keys can provide more robust identifications because two users, choosing slightly different character states, could arrive at the same result. Multi‐access keys, however, are still susceptible to user interpretation of which character state to choose. One disadvantage is that a multi‐access key may not always result in a single annotation. In an instance where more than one taxon results, the user must either select one of the taxa based on unquantified or arbitrary judgment, or place the unknown in the next higher taxon (genus instead of a species, for example). The latter has the undesirable effect of reducing the precision of the identification while the former potentially reduces the accuracy. The method presented here extends the benefits of multi‐access identification keys by accounting for the uncertainty of both the species (as determined by the author) and the user in a structured and repeatable way. The author builds the degree of certainty of a character into the key by adjusting the spread of the underlying distribution, while the user can specify their uncertainty by adjusting the precision of their measurement (equating to the width of the integral under the probability density function). Both of these will feed into the resulting posterior probability for a particular taxon or set of taxa.

The NB assumption simplifies implementation of the classifier, but it also introduces the restriction that characters must be independent. Taking care to choose characters that have little correlation in order to meet this assumption is not an unreasonable trade‐off for the benefit. When correlated characters are necessary, an extension such as augmented NB (Friedman et al., 1997) could be used to relax the independence assumption.

The NB classifier can blend both continuous characters and traditional multistate characters by calculating probabilities for multistate character states from uniform‐discrete probability mass functions. The probability of each state would simply be 1/*n*, where *n* is the number of states expressed by a taxon. For example, the probabilities for “leaves cordate or deltoid” would be: cordate = 0.5, deltoid = 0.5. Discrete characters, even in an NB context, suffer some of same criticisms directed at other methods. An author of a key, however, could still adjust the certainty in or variability of the character states. Variability in the frequency of the character states is incorporated by weighting the discrete states for a taxon as a proportion of the total. For example, perhaps a species has “leaves usually cordate, sometimes deltoid.” This could be weighted as 19 in 20 are cordate and 1 in 20 is deltoid, with the resulting probability for each state being: cordate = 0.95, deltoid = 0.05. Of course, the more complex the model, the more the onus is on the author of the key to encode all of these features. On the other hand, even characters considered to be discrete might be describable as a spectrum (e.g., shades of color [wavelength], hairiness [hairs/cm^2^], relative position [e.g., mm to/from]).

The majority of published taxonomic data for quantitative characters are reported as extrema values (minimum‐maximum sets). Without a liberal application of assumptions, these data are not readily amenable to generating the center and spread statistics required to estimate non‐uniform probability distributions. Normally distributed characters require only a few assumptions to coerce range data into something approximating first‐ and second‐order moments, but most morphological characters with minima equal or close to zero fail to be normally distributed and are better suited to a truncated distribution or a distribution that is limited to a positive domain (e.g., gamma, log‐normal). These distributions cannot be coerced from range data. Data transformation to approach normality is a possibility, but the algorithm presented here would have to be modified to transform the user input in an identical manner to the model data in order to correctly calculate the probabilities.

Characters with minima much greater than zero are also not safely assumed to be normally distributed. Published species accounts sometimes include characters with unusually low or high values often bracketed with parentheses, for example: 90–100(–200). This could be an indicator of a character that conforms to a non‐symmetric or complex probability density function. It could also be caused by the lumping of one or more undiagnosed taxa, each with symmetric character distributions, into a single species class. Regardless, having only extrema data does not allow the author of a key to visualize or test the distribution of character states for a taxon and presents a barrier to adapting traditional species information to the NB classifier framework presented here.

Actual character data are not always normally distributed (Henderson, 2002, 2004), so normality cannot always be assumed. If an author strongly believes that a character is normally distributed but lacks the training data to calculate the means and standard deviations for all species, one possibility would be to coerce these values from the range extrema by: (1) assuming the median and mean are equal and substituting the former for the latter using the method of moments, then (2) assuming that the character state range is analogous to some credibility interval (e.g., 99%) and solving for standard deviation by substituting the minimum and maximum values as the interval's quantiles. This is not a very satisfactory way of leveraging conventional data, however. Unless morphological data sets are gathered that are large enough to reliably assess the shape of a character's distribution and generate estimates of its moments, implementation of the quantitative key method outlined here will need to rely on either uniform distributions or assumed center and spread statistics.

Modern taxonomy is often built on a lineage‐based species concept (de Queiroz, 2007), but these do not guarantee a morphological diagnosis is included. Until genetic analysis tools are accurate and feasible enough for field use, practical species identifications in industry and resource management sectors will still use a morphological approach. Organism phenotypes are a rich, multidimensional source of data for identifications, but current methods for identification keys truncate their underlying potential and limit an end user's ability to assess the accuracy of the species class to which they assign an individual. The NB classifier I have presented, while not new, has yet to be adopted by taxonomists.

### Future directions

I have shown that the NB classifier holds promise in its ability to make use of the rich, complex, and probabilistic nature of continuous phenotypic data—a data source that is becoming more accessible with international efforts to digitize herbarium specimens (e.g., Unger et al., 2016). I suggest that software developers incorporate this method into existing technologies or create new applications so that the authors of interactive taxonomic keys have a platform for creating keys with probabilistic identifications. Until these applications are created, researchers with computer programming experience can create scripts for exploratory analyses of difficult taxa, in‐house identification keys, or even deployment of interactive web apps with packages such as *R Shiny* (Chang et al., 2018). To facilitate this, I have provided an annotated script (Appendix S1) containing all the examples in this study that is executable using R (R Foundation, 2018). My recommendation to researchers doing revisionary taxonomic work is that we begin examining the measurement distributions for the continuous characteristics of specimens and report either our raw data or appropriate summary statistics in addition to the minima and maxima. This will lay the foundation for better identification tools that can leverage the full power of continuous morphological characters.

## Supporting information


**APPENDIX S1.** Script in R (R Foundation, 2018) demonstrating the results and examples used in this study.

**APPENDIX S2.** Normal quantile‐quantile (Q‐Q) plots for the species and hybrid of *Myriophyllum* from example 1. Large deviations from the diagonal line suggest that a normal probability density function may not provide accurate posterior probabilities. (A) *Myriophyllum spicatum*, (B) *M. sibiricum*, and (C) *M. sibiricum* × *M. spicatum* hybrid.

## Data Availability

Data and R scripts used here are available at https://github.com/cdtyrrell/id-key.

## APPENDIX 1 A general algorithm for using a naive Bayes process to find the posterior probabilities that an unknown specimen with character measurements *m* and measurement precision (error) *e* is attributable to a taxon. The classifier takes as input matrix *M* (means) and *S* (standard deviation [or variance depending on density function]), for each of *n* characters and *k* taxa with normally distributed characters.

**Table:**

**input:**	
matrix M[k, n], S[k, n]	
vector m[n], e[n]	
**output:**	
vector result[k]	
1:	pr <‐ **log**(1/k) # uniform prior
2:	**for each** row **in** M **do**
3:	**for each** column **in** M **do**
4:	q1 <‐ m[column] ‐ e[column] # calculate quantiles
5:	q2 <‐ m[column] + e[column]
6:	area <‐ approx. definite integral of
	Normal(mean = M[row, column], sd = S[row, column])
	for the interval (q1, q2)
7:	pr <‐ pr + **log**(area)
8:	result[row] <‐ **exp**(pr)
9:	**return** result

## APPENDIX 2 Leaf segment (pinnae) number from approximately 109 specimens of *Myriophyllum spicatum*,* M. sibiricum*, and their interspecific hybrid estimated from Fig. 4 of Moody and Les (2007).

**Table:**

No. of segments per leaf	Frequency of occurrence		
	*M. sibiricum*	*M. spicatum*	Hybrid
9	1	0	0
10	2	0	0
11	4	0	0
12	2	0	0
13	2	0	0
14	3	0	0
15	12	0	0
16	14	0	1
17	17	0	1
18	14	0	2
19	18	0	10
20	8	0	10
21	14	0	11
22	8	0	11
23	4	1	19
24	0	1	27
25	0	2	30
26	0	6	32
27	1	9	45
28	0	14	26
29	0	16	28
30	0	22	11
31	0	23	21
32	0	29	7
33	0	26	6
34	0	26	7
35	0	17	8
36	0	12	3
37	0	5	3
38	0	6	2
39	0	1	1

## APPENDIX 3 Means and standard deviations (SD) for leaf width (mm) and number of branches per node among four species of *Rhipidocladum* native to Mexico. Moments are calculated from unpublished herbarium specimen measurements.

**Table:**

Species	Sample size	Leaf width (mm)		No. of branches per node	
		Mean	SD	Mean	SD
*R. bartlettii* (McClure) McClure	19	7.17	1.53	34.14	29.3
*R. martinezii* Davidse & R. W. Pohl	3	4.00	0.50	150.0	25.0
*R. pittieri* (Hack.) McClure	45	10.60	4.94	24.71	10.0
*R. racemiflorum* (Steud.) McClure	63	5.18	1.48	43.05	28.5
