# Supplementary material for: A method to implement continuous characters in digital identification keys that estimates the probability of an annotation
Source: Appl Plant Sci. 2019 May 8;7(5):e01247. doi: 10.1002/aps3.1247 (PMC6526653; doi:10.1002/aps3.1247)

Tyrrell—Applications in Plant Sciences 2019 7(5)—Data Supplement S2
DOI: 10.1002/aps3.1247

APPENDIX S2. Normal quantile-quantile (Q-Q) plots for the species and hybrid of *Myriophyllum* from example 1. Large deviations from the diagonal line suggest that a normal probability density function may not provide accurate posterior probabilities. (A) *Myriophyllum spicatum*, (B) *M. sibiricum*, and (C) *M. sibiricum* × *M. spicatum* hybrid.

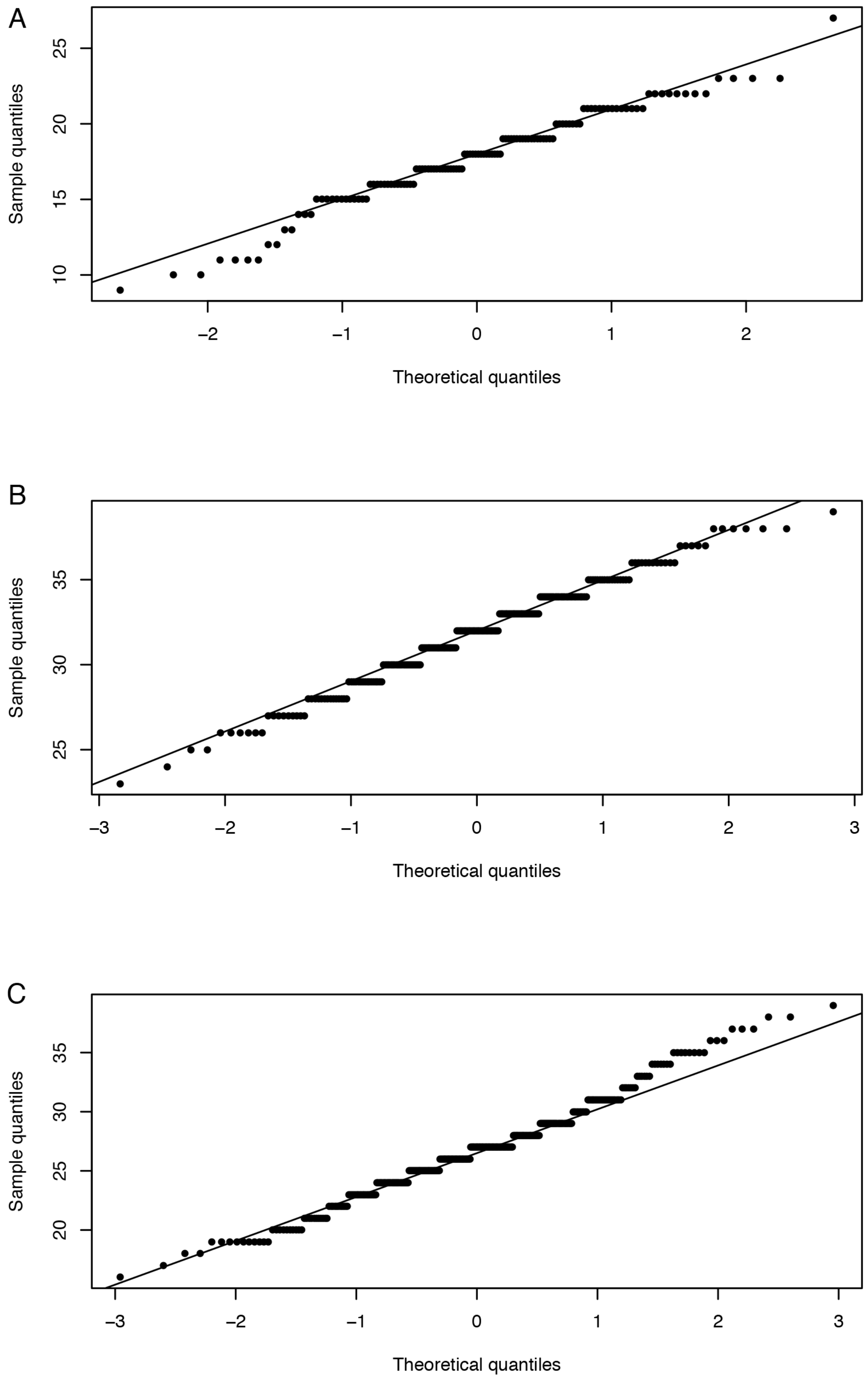

Supplement: Supplementary file 2 — APPENDIX S2. Normal quantile‐quantile (Q‐Q) plots for the species and hybrid of Myriophyllum from example 1. Large deviations from the diagonal line suggest that a normal probability density function may not provide accurate posterior probabilities. (A) Myriophyllum spicatum, (B) M. sibiricum, and (C) M. sibiricum × M. spicatum hybrid. [file APS3-7-e01247-s002.pdf]
